# Guiding dose adjustment of amlodipine after co-administration with ritonavir containing regimens using a physiologically-based pharmacokinetic/pharmacodynamic model

**DOI:** 10.1007/s10928-018-9574-0

**Published:** 2018-02-09

**Authors:** Dwaipayan Mukherjee, Jiuhong Zha, Rajeev M. Menon, Mohamad Shebley

**Affiliations:** Clinical Pharmacology and Pharmacometrics, AbbVie Inc., 1 North Waukegan Road, Dept. R4PK, Bldg. AP31-3, North Chicago, IL 60064 USA

**Keywords:** PBPK, Amlodipine, Systolic blood pressure, CYP3A4, Dose adjustment, Ritonavir

## Abstract

Amlodipine, a commonly prescribed anti-hypertensive drug, shows increased systemic exposure with cytochrome P450 (CYP) 3A inhibitors. Ritonavir (RTV) is a potent mechanism-based and reversible CYP3A inhibitor and moderate inducer that is used as a pharmacokinetic enhancer in several antiviral treatment regimens. Drug–drug interaction (DDI) between RTV and amlodipine is due to mixed inhibition and induction of CYP3A4, which is challenging to predict without a mechanistic model that accounts for the complexity of both mechanisms occurring simultaneously. A novel physiologically-based pharmacokinetic (PBPK) model was developed for amlodipine, and the model was verified using published clinical PK and DDI data. The verified amlodipine PBPK model was linked to a pharmacodynamics model that describes changes in systolic blood pressure (SBP) during and after co-administration with RTV. The magnitude and time course of RTV effects on amlodipine plasma exposures and SBP were evaluated, to provide guidance on dose adjustment of amlodipine during and after co-administration with RTV-containing regimens. Model simulations suggested that the increase in amlodipine’s plasma exposure by RTV diminishes by approximately 80% within 5 days after the last dose of RTV. PBPK simulations suggested that resuming a full dose of amlodipine [5 mg once daily (QD)] immediately after RTV’s last dose would decrease daily average SBP by a maximum of 3.3 mmHg, while continuing with the reduced dose (2.5 mg QD) for 5 days after the last dose of RTV would increase daily average SBP by a maximum of 5.8 mmHg. Based on these results, either approach of resuming amlodipine’s full dose could be appropriate when combined with appropriate clinical monitoring.

**Electronic supplementary material:**

The online version of this article (10.1007/s10928-018-9574-0) contains supplementary material, which is available to authorized users.

## Introduction

The calcium-channel blocker amlodipine is a common anti-hypertensive medication, and was the fifth most prescribed drug in the United States (US) with 57.2 million prescriptions in 2010 [1]. Approximately 50 million adults in the US suffer from hypertension, and for those with co-morbidities, there is a potential for DDI due to polypharmacy [2]. Amlodipine is primarily metabolized and cleared from the body by the drug metabolizing enzyme CYP3A4, with a lesser (10%) contribution from CYP3A5 [3]. Therefore, co-administering amlodipine with drugs that are strong inhibitors of CYP3A4 may increase the plasma concentrations of amlodipine, where monitoring for symptoms of hypotension and edema in patients become necessary [4]. RTV is a strong CYP3A4 inhibitor and is a used as a pharmacokinetic enhancer in drug combinations with other anti-retrovirals and protease inhibitors such as indinavir, lopinavir, paritaprevir and darunavir [5, 6]. Concomitant administration of RTV-containing regimens with amlodipine may lead to an unintended increase in the plasma exposure of amlodipine and a subsequent drop in blood pressure. The prescribing labels for the direct-acting antiviral (DAA) regimens for the treatment of chronic hepatitis C virus infection, ombitasvir/paritaprevir/RTVplus dasabuvir (3-DAA regimen) [5] or ombitasvir/paritaprevir/RTV (2-DAA regimen) [7] recommend dose reduction of amlodipine by 50% and to monitor patients for clinical effects of amlodipine. Similarly, the United States Prescribing Information (USPI) for lopinavir/RTV calls for “caution” and “clinical monitoring” when dosed with dihydropyridine calcium-channel blockers which are dependent on CYP3A for metabolism [8]. RTV is a known time-dependent inhibitor and inducer of CYP3A4, and at steady state a net inhibitory effect is observed with sensitive CYP3A substrates [9, 10]. RTV increased amlodipine plasma exposure (AUC) by 89% (as a combination with indinavir [11]) and 157% (ombitasvir/paritaprevir/RTV plus dasabuvir [12]). Effects of time-dependent inhibition and induction can outlast RTV’s last administered dose and plasma concentrations, until turnover of endogenous CYP3A4 allows the enzyme levels to reach homeostasis again. This raises a question about the duration of the net inhibitory effect on CYP3A4 after the RTV-containing therapy is stopped and when the standard dose of amlodipine can be resumed, which cannot be directly extrapolated due to the complex nature of the reversible and mechanism-based inhibition and induction by RTV.

PBPK models can be of great value for simulation of the various dose regimens and analysis of the dynamic change in plasma concentrations over time for the victim and inhibitor drugs, and relative to the physiological change in abundance of CYP enzymes due to mechanism-based inhibition or induction. These models have been used frequently to simulate drug pharmacokinetics [13–15] and especially in elucidating complex DDI with various co-medications, all of which may be impossible to study through dedicated clinical trials [16–18]. PBPK models are often linked with PD models in order to predict changes in drug effect due to extrinsic or intrinsic factors that affect the drug PK, for which a recent example being the work of Moj et al. [19]. Blei [20] developed a PBPK model for amlodipine, but the model did not include CYP3A4-mediated clearance, which is essential in order to model mechanistic DDI with perpetrator drugs. Dennison et al. [21], developed a PBPK model for amlodipine which included CYP3A4-mediated clearance, however their focus was on dissolution and oral absorption of amlodipine. Dennison et al. also assigned the entire oral clearance to be due to CYP3A4 and there was no effort to confirm the contribution of the particular enzyme through DDI with CYP3A4 inhibitors.

The development and verification of a novel PBPK model for amlodipine, and its application in simulating various dose regimens in the presence or absence of RTV are described in this article. The amlodipine PBPK model was linked to a pharmacodynamic (PD) model, which described changes in SBP, in order to inform a clinically-relevant dose adjustment for amlodipine during and after co-administration with RTV-containing therapies, e.g., the 3-DAA or 2-DAA regimen.

## Methods

### Amlodipine PBPK model development

A novel PBPK model was developed for amlodipine using information from the literature. Amlodipine is a dihydropyridine base (pKa = 9.1) [22] and a highly soluble compound (solubility = 0.774 mg/mL) [23]. Systemic clearance of amlodipine is primarily mediated by the CYP3A4 enzyme, although a small contribution (10%) from CYP3A5 has been reported [24]. The contribution of renal clearance in the disposition of amlodipine has been reported to be only 6% [25]. The PBPK model for amlodipine was developed in Simcyp^®^ version 15R1 simulator (Certara Inc.). The Simcyp software platform [26, 27] has been widely used for PBPK modeling and simulation of pharmaceutical compounds by multiple commercial and academic groups, as well as regulatory agencies. Amlodipine physicochemical properties (logP, pKa, molecular weight) and absorption, distribution, metabolism and elimination (ADME) parameters are summarized in Table 1. The ADME parameter values obtained from literature were used as initial estimates in the PBPK model, and a first-order absorption model within Simcyp^®^ was selected based on the available information. Amlodipine is a BCS class I compound with high solubility and permeability [23, 28], and absorption rate (*k*_*a*_) has been reported by Flynn et al. [29]. Stopher et al. [30] found based on a human mass balance study that the entire amount of amlodipine administered orally is absorbed, which suggests that fraction absorbed (*f*_*a*_) is 1. Despite its high solubility, amlodipine is reported to have a prolonged oral absorption as suggested by its long time to maximum plasma concentration (T_max_, 6–9 h) [31]. This was captured by optimizing the *k*_*a*_ and absorption lag time (*t*_*lag*_) based on the observed plasma profile after oral administration [32]. The final optimized value of *k*_*a*_ = 0.75 was very close to that reported by Flynn et al. [29]. Distribution of amlodipine within the body was modeled using a ‘minimal PBPK model’ available within Simcyp, which models distribution using a central compartment (volume = V_d_) and a single adjusting compartment (SAC) (volume = V_SAC_, blood flow rate = Q_SAC_). Hepatic distribution and blood flow is also considered separately. Tissue distribution was estimated using tissue-plasma partition coefficients which are estimated in Simcyp V15 using the method of Poulin and Theil [33], corrected by Berezhkovsky et al. [34] Other parameters of the model were optimized using ‘top-down’ optimization based on published clinical PK data (plasma concentrations) of amlodipine in healthy volunteers. Clinical data for model optimization were obtained from Faulkner et al. [32], which comprised mean plasma concentration data for 12 healthy volunteers after intravenous (IV) and oral doses of amlodipine. The PBPK model was optimized using the ‘fasted’ state for the oral dose and the IV dose (constant rate IV infusion of 10 min) in accordance with the clinical study design by Faulkner et al. [32]. For parameter optimization within Simcyp V15, the Nelder-Mead algorithm with the sum of weighted least squared errors as the objective function was used. Since CYP3A4 is the major elimination pathway for amlodipine and is the cause for DDI with various CYP3A perpetrator drugs, amlodipine intrinsic clearance parameters for CYP3A4 were further optimized using clinical DDI data (C_max_ and AUC ratios). The DDI study that was used for optimization involved co-administration of amlodipine with a combination of indinavir and RTV in 18 healthy, HIV-seronegative adults. In the study by Glesby et al., [11] amlodipine was dosed alone at 5 mg QD for 7 days initially. This was followed by dosing of indinavir + RTV (800 mg twice daily [BID]/100 mg BID) for 19 days (Day 8–Day 26) and amlodipine (5 mg QD) on Day 20–Day 26. Plasma PK was measured on Day 7 and again on Day 26. A schematic representation of the study design is included in the supplementary information (Figure S1). A Comparison of PK parameters (maximum plasma concentration, C_max_, and area under the plasma-concentration time curve from time 0 to 24 h, AUC_24_) on Day 7 and Day 26 was carried out to quantify the effect of steady-state RTV on steady-state levels of amlodipine. The PBPK model simulated a trial design identical to the original clinical study by Glesby et al. [11]. A PBPK model for RTV was developed by Shebley et al. [35] incorporating reversible, time-dependent and mechanism-based inhibition and induction of CYP3A4. The RTV PBPK model was used as reported in Shebley et al. for the purpose of simulating DDI with amlodipine. The contribution of CYP3A4 to amlodipine clearance was optimized by optimizing the values of *V*_*max*_ and *K*_*m*_ for CYP3A4 and by assigning additional clearance to biliary and non-specific pathways. Simcyp^®^ incorporates population characteristics for healthy volunteers and includes observed population distributions of physiological parameters including tissue volumes, blood flow rates, metabolizing enzyme abundances, etc. [26]. These values have been obtained from multiple references and are summarized by multiple Simcyp-specific publications. [36–38]. Enzyme expression and turnover values for specific enzymes including CYP3A4/3A5 are built-in within the Simcyp simulator and these have been verified/validated in multiple instances separately [39, 40]. The “population representative” virtual healthy volunteer was used within Simcyp^®^ for the optimization of model parameters.

**Table 1:** Physicochemical properties and parameters included in the physiologically-based pharmacokinetic (PBPK) model for amlodipine

Property/parameter	Value	Source/method
Initial estimates		
Mol. weight	408.88 g/mole	www.drugbank.ca
Fraction unbound (plasma)	0.025	Drug label [4]
logP (n-octanol:water)	2.96	Caron et al. [22]
Blood:Plasma ratio	0.596	Simcyp^®^ prediction toolbox
pKa (base)	9.1	Caron et al. [22]
Absorption rate constant	k_a_ = 0.8 h^−1^	Flynn et al. [29]
Fraction absorbed	f_a_ = 1	
Volume of distr.	V_d_ (IV) = 21.4 L/kg	Faulkner et al. [32]
Vol. of SAC	V_SAC_ = 6.38 L/kg	Park et al. [41]
Flow rate into SAC	Q_SAC_ = 102 L/h	
Metabolism	CYP3A4 (10% CYP3A5)	Zhu et al. [24]
Elimination	6% renal clearance	Beresford et al. [25]
	33.9 L/h (IV clearance)	Faulkner et al. [32]
Optimized parameters		
Absorption rate constant	0.75 h^−1^	Optimized based on oral PK
Absorption lag time	3.2 h	
Fraction unbound in gut	0.2	
V_SAC_	11 L/kg	
Q_SAC_	90 L/h	
CYP3A4 intrinsic clearance	CL_int_ = 170 L/h	Optimized based on oral PK and DDI
CYP3A5 intrinsic clearance	CL_int_ = 43.5 L/h	Estimated relative to CYP3A4^a^ [24]
Biliary clearance	12 L/h	Optimized based on oral PK
Renal clearance	1.8 L/h	Based on 6% of total CL [25]
Non-specific clearance	16 L/h	Optimized based on oral PK

*SAC* single adjusting compartment

^a^Based on intrinsic clearance due to CYP3A4 and relative contribution of CYP3A5 and also the relative differences in the abundances of CYP3A4 and CYP3A5 in the gut and in the liver

### Amlodipine PBPK model verification

The PBPK model was verified against data (plasma concentrations) from the oral multiple dosing arm in the study by Faulkner et al. [32] involving healthy volunteers. Amlodipine was administered at 15 mg QD for 14 days to 28 healthy subjects. The developed PBPK model was verified using 3 external verification data sets (comprising plasma concentration values) of amlodipine PK (summarized in Table 2) [28, 42, 43]. For verification, the PBPK model was used to simulate plasma concentrations in 100 virtual individuals (10 trials of 10 subjects each). The model was additionally verified using clinical DDI data (C_max_ and AUC ratios) with RTV (ombitasvir/paritaprevir/RTV + dasabuvir) from Menon et al. [12]. This study involved an amlodipine single dose of 5 mg on day 1, followed by a 10 day washout, RTV multiple dose administration of 100 mg QD and then another amlodipine dose of 5 mg at RTV steady state. Schematic representations of the study design have been included in the supplementary information (Figure S1). The model acceptance criterion was pre-specified as a 20% prediction error relative to the observations, consistent with the bioequivalence criteria (80–125%) for PK metrics [44]. This means that the ratio of the model predicted value of a PK parameter (C_max_ or AUC) to the observed PK parameter value must fall within the 0.8–1.25 range to be acceptable. A local sensitivity analysis of the final PBPK model was also carried out, considering C_max_ and 24 h AUC as the output variables. Parameters of the PBPK model were varied from 0.1 to 10 fold of their nominal values using the sensitivity analysis tool within Simcyp V15.

**Table 2:** Summary of published clinical studies used for obtaining mechanistic information and for model optimization and validation

Study	Population	Information/Results	Source
IV and oral single dose PK study	12 healthy subjects	PK parameters optimized	Faulkner et al. [32]
Renal impairment study	27 renally impaired subjects	No significant changes	Laher et al. [45]
IV and oral^14^C study	2 healthy subjects	Renal clearance optimized	Beresford et al. [25]
DDI study with Indinavir + RTV	18 healthy HIV-negative subjects	Model optimization	Glesby et al. [11]
Multiple oral dose study	28 healthy subjects	Model verification	Faulkner et al. [32]
PK study with increasing doses of amlodipine	12 healthy subjects	Model verification	Williams and Cubeddu [43]
Amlodipine PK study	24 healthy subjects	Model verification	Rausl et al. [28]
Food effect study	6 healthy subjects	Model verification	Faulkner et al. [42]
DDI study with Viekira Pak	14 healthy subjects	Model verification	Menon et al. [12]
PopPK/PD study for amlodipine	73 subjects with hypertension	Direct-effect PD model development	New drug application for amlodipine [3]
6 week PK/PD study	12 subjects with hypertension	Indirect-effect PD model development	Donnelly et al. [46]

*IV* intravenous, *PK* pharmacokinetic, *DDI* drug–drug interaction, *RTV* ritonavir, *popPK* population pharmacokinetic, *PD* pharmacodynamic

### Amlodipine PBPK model application: RTV DDI prediction

The developed and verified PBPK model was used to simulate multiple dosing of amlodipine when co-administered with the RTV-containing 3-DAA regimen [5]. As RTV is the only clinical inhibitor and inducer of CYP3A4 within the regimen, only RTV was simulated as a surrogate for the DAA regimen. Amlodipine at 5 mg QD alone was simulated for 14 days to reach steady state, followed by the combination of 100 mg QD RTV and 2.5 mg QD amlodipine for an additional 14 days. The 50% reduced amlodipine dose was in accordance with labelling recommendations for the DAA regimens. After this 28-day schedule, 2 different dose regimens were simulated. In the first regimen, amlodipine at a reduced dose (2.5 mg QD) was continued for 5 additional days after the last dose of RTV, followed by a return to a full (5 mg QD) dose of amlodipine. In the second regimen, the regular dose of amlodipine (5 mg QD) was resumed immediately after the last dose of RTV.

### Amlodipine PD model development

A PD model that describes the effects of amlodipine on SBP was developed to link the dynamic plasma concentration of amlodipine with SBP, in order to understand the effect of DDI on clinical outcomes. Studies have shown that it is more important to control SBP than diastolic blood pressure [47], and SBP was found to be the best single predictor of cardiovascular disease and coronary heart disease in multiple trials [47–49]. Amlodipine is a calcium-channel blocker that causes systemic vasodilation, helping in the management of hypertension. Due to the long half-life of amlodipine (~ 40 h), a single dose of amlodipine is effective in reducing SBP over 24 h [50]. Thus SBP was selected as the endpoint in the PD model. It is also well known that blood pressure along with heart rate has a pronounced circadian rhythm, characterized by substantial reductions during sleep, a rapid increase after awakening, and variability during the day [51, 52]. The diurnal variation is an important consideration in the clinical management of hypertension and cardiovascular disease [52] and hence it was included in the PD model as a baseline effect.

Two previously published PD models for amlodipine were initially considered: a direct-effect model [3] and an indirect-effect model [46]. The direct-effect model is a regression equation model based on clinical observations for amlodipine [3], and it relates SBP to daily average plasma concentrations of amlodipine. The indirect-effect model developed by Donnelly et al. [46] considers an additional effect compartment that takes into account the delay between amlodipine plasma exposure and the lowering of blood pressure (Eq. 1):

1 $$SBP = SBP_{0} + m.C.\exp ( - k_{eo} t)$$

where, *SBP*_*0*_ is the baseline systolic blood pressure in mmHg, *C* is the dynamic plasma concentration of amlodipine in ng/mL, *k*_*eo*_ is the elimination rate constant from the effect compartment, *t* is time after first dose in hours, and *m* is a first-order rate constant. The indirect-effect model, which uses dynamic plasma concentration, was used to model the effect of amlodipine on changing SBP. The model was fitted to mean SBP from 12 hypertensive patients reported by Donnelly et al. [46]. *SBP*_*0*_ is not constant and has significant diurnal variability, as discussed earlier. Circadian rhythms in baseline models have been developed by Sällström et al. [53] for body temperature, heart rate, and blood pressure regulation, where oscillatory functions have been used to capture dynamic changes in the baseline value of the relevant variable. Accordingly, a cosine function was fitted to the clinical SBP data in the placebo and drug arms from Donnelly et al. [46], with a time shift as shown in Gabrielsson and Weiner [54]. The dynamic baseline model developed here can be represented as Eq. 2:

2 $$SBP_{0} = P_{0} + A\cdot\cos \left( {\frac{2\pi f}{24}T} \right)$$

where, *P*_*0*_ is the initial SBP at the beginning of the day, *A* is the amplitude of the circadian variation, *f* is the frequency of SBP oscillation, T is the 24-h clock time and is calculated as: T = time after first dose—number of full days elapsed × 24. The parameters in Eq. (2) were estimated by fitting the equation to clinical SBP measurements after administration of placebo as described by Donnelly et al. The fitting was performed using MATLAB 2016b (using the fminsearch function which uses the Nelder-Mead optimization algorithm) and the final fitted parameters are summarized in Table 3. The parameters in Eq. (1) were fitted using clinical SBP measurements at Day 1 and Day 43 after a daily dose of 5 mg of amlodipine as described by Donnelly et al. The final fitted parameters are summarized in Table 3. It should be noted that the PD parameters used in the indirect-effect model were fitted to a hypertensive population data as described by Donnelly et al. [46] and there was high inter-individual variability associated with parameter values *m* and *k*_*eo*_. Since only the mean observations were reported in Donnelly et al., the model parameters were fitted to the mean data only. The complete PD model combining Eqs. (1) and (2) can be represented as follows:

3 $$SBP = P_{0} + A\cos \left( {\frac{2\pi f}{24}T} \right) + mC\exp ( - k_{eo} t)$$

**Table 3:** Fitted parameter values for the pharmacodynamics model for amlodipine

Parameter	Symbol	Value	Residual error^c^
Baseline model^a^			
Initial pressure	*P*_*0*_	148.8 mmHg	2.89
Circadian amplitude	*A*	8.25 mmHg	
Circadian frequency	*f*	1.76 day^−1^	
Drug effect model^b^			
Direct effect rate	*m*	− 3.145 mmHg·mL/ng	5.04
Indirect effect rate constant	*k*_*eo*_	0.049 h^−1^	

^a^Baseline circadian rhythm model for SBP

^b^Drug effect model on SBP

^c^Residual error estimated as $\sqrt {\frac{{\sum {\left( {y - y^{\prime}} \right)^{2} } }}{N}}$, where y and y’ represent the observed and predicted values of SBP respectively

The PD model (shown in Eq. (3)) was implemented in Simcyp^®^ V15 in order to form an integrated PBPK/PD model. The PD model was incorporated as a ‘custom PD’ module using the *Lua* scripting feature in Simcyp^®^ as described in Abduljalil et al. [55]. The relevant *Lua* script is provided in the supplementary information as Figure S2.

### Amlodipine PBPK/PD model application

The developed PBPK/PD model was used to simulate the changes in SBP due to the dynamic changes in amlodipine plasma exposures with and without RTV. Three clinical scenarios were simulated for comparing the effect on SBP. In scenario 1, model simulations were carried out with RTV, with and without dose adjustment of amlodipine, to understand the effect of dose adjustment on SBP. Amlodipine PK was simulated after a regular dose of 5 mg QD and a RTV dose of 100 mg QD starting on day 14 and continuing for 14 days. In one simulation, the amlodipine dose was continued at 5 mg QD during coadministration with RTV. In the other simulation, the amlodipine dose was reduced by 50% (to 2.5 mg QD) during coadministration with RTV. In scenario 2, two simulations of dose adjustment were performed. In the first simulation, the reduced amlodipine dose of 2.5 mg QD was continued for 5 additional days after the last dose of RTV. In the other simulation, the regular amlodipine dose was resumed immediately after RTV was stopped. Scenario 3 is similar in design to scenario 2, but the effect of a higher amlodipine dose (10 mg QD, the maximum dose per the USPI [4]) was simulated. All model simulations were carried out using a virtual population representative within Simcyp^®^.

## Results

### Single and multiple dose pharmacokinetics of amlodipine

**Fig. 1:**
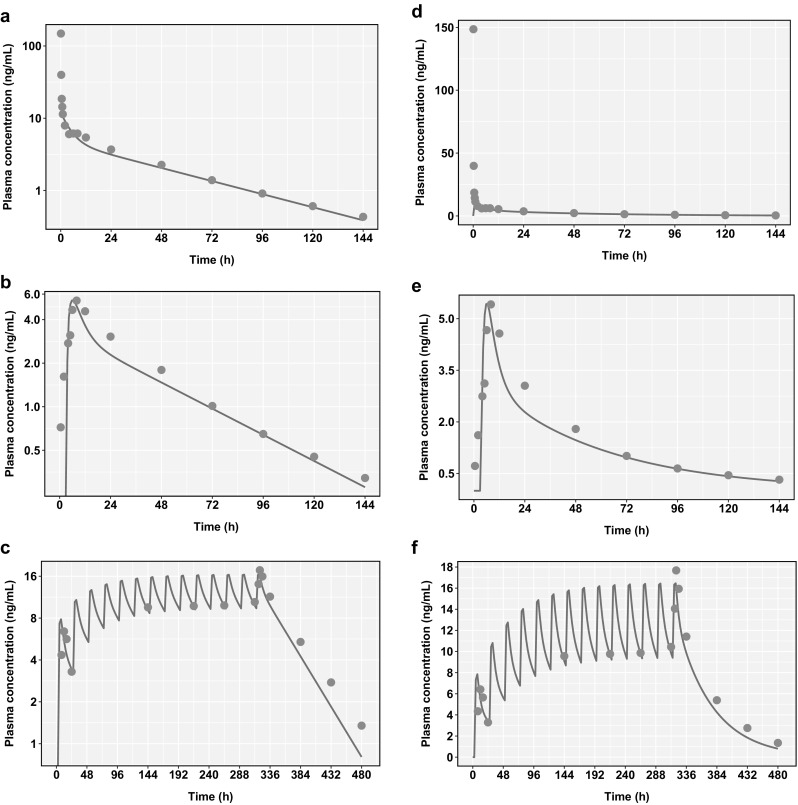
Comparison of physiologically-based pharmacokinetic (PBPK) model predictions (blue lines) of plasma concentrations of amlodipine after **a** a 10 mg intravenous (IV) infusion, **b** a 10 mg oral dose, and **c** multiple amlodipine dosing of 15 mg once daily (QD) for 14 days. The model shows plasma concentrations to reach steady state in about 7 days. **d**, **e**, and **f** represent the same plots with linear y-axes. Clinical data (mean of 12 subjects for single dose studies and 28 subjects for the multiple dose study) from Faulkner et al. [32] are represented as orange dots (Color figure online)

**Fig. 2:**
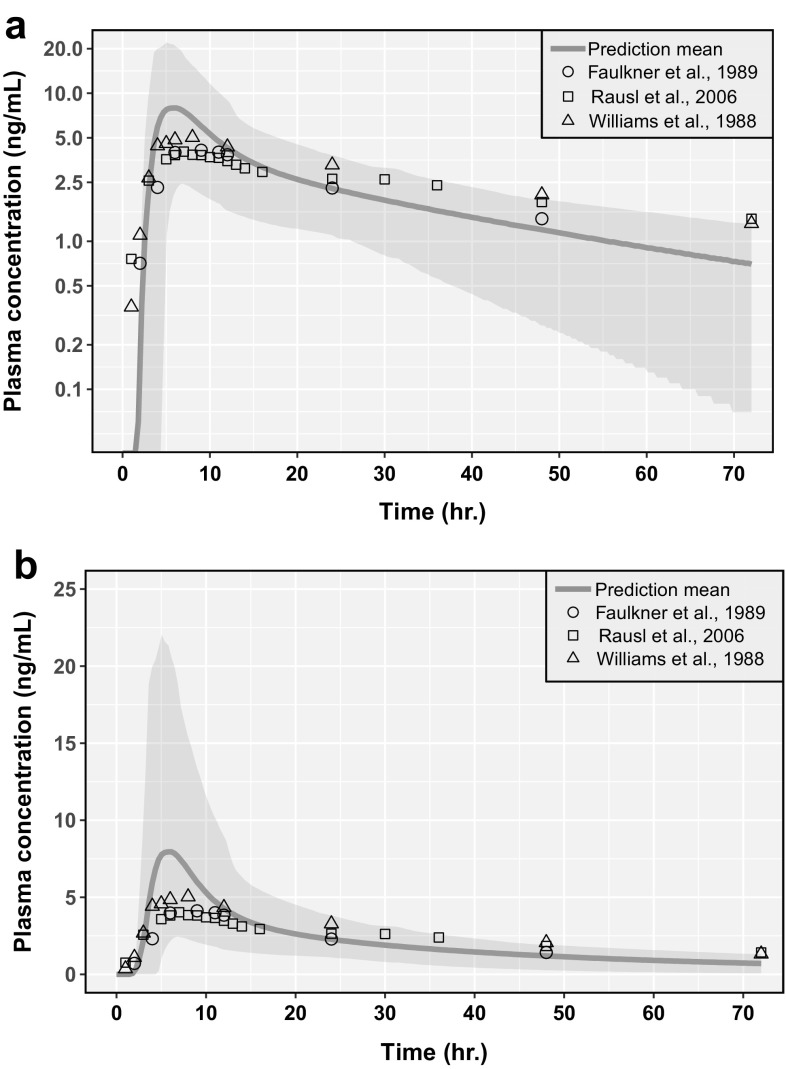
Comparison of physiologically-based pharmacokinetic (PBPK) model predictions of 100 virtual subjects (10 trials of 10 subjects each) with clinical observations across multiple studies after a single amlodipine oral dose of 10 mg, shown with a log y-axis (**a**) and a linear y-axis (**b**). The Y axis shows the plasma concentrations of amlodipine. The red line represents the prediction mean and the red shaded area represents the 5th–95th percentiles of the predictions (Color figure online)

The optimized PBPK model resulted in a good agreement between observed and predicted values for amlodipine IV (Fig. 1a) and oral (Fig. 1b) PK profiles after single dose administration. The model predictions of amlodipine C_max_ and area under the concentration–time curve extrapolated to infinity (AUC_∞_) were within an 18% prediction error as shown in Table 4, and consistent with the pre-specified acceptance criterion of a 20% prediction error. Figure 1c shows the simulated amlodipine concentration–time profile following multiple dose administration of amlodipine alone at 15 mg QD (14 doses) with plasma concentrations measured over 20 days (multiple dose study from Faulkner et al. [32]). The predicted plasma concentration of amlodipine reached steady state at around 7 days, consistent with the reported observations [31]. The PBPK model predicted an accumulation ratio for C_max_ (Day 14:Day1) of 2.1 compared with the observed value of 2.6 (observed range 0.9–5.7), and an accumulation ratio of the minimum plasma concentration (C_min_) (Day14:Day1) of 2.9, compared with the observed value of 3.6 (observed range 1.6–11.7). The model-predicted accumulation ratio of AUC_24_ (Day14:Day1) was 2.9, compared with the observed value of 3.2 (observed range 1.2–7.4). Figure 2 shows the model predictions of 10 trials each consisting of 10 virtual subjects. The 5 and 95% predicted percentiles of the plasma concentrations included all of the observed data from 3 different clinical trials. The PBPK model predictions of amlodipine plasma concentrations following single and multiple dosing were consistent with the clinically observed data and met the model acceptance criteria. Sensitivity analysis results from a local sensitivity analysis are presented in the Supplementary Information (Figure S6).

**Table 4:** Comparison of model predicted and clinically observed pharmacokinetic parameters (Results are for a population representative virtual subject for all studies used for optimization, except Menon et al., which is verification in a virtual population of 100 subjects)

Clinical study	PK parameter	Prediction	Observation	Pred:obs Ratio
Faulkner et al. (IV) [32]	AUC_∞_ (ng-h/mL)	303	371	0.82
	t_1/2_ (h)	39.9	33.8	1.18
Faulkner et al. (oral) [32]	AUC_∞_ (ng-h/mL)	201	238	0.84
	C_max_ (ng/mL)	5.45	5.9	0.92
	T_max_ (hr.)	6.09	7.6	0.8
	t_1/2_ (h)	39.9	35.7	1.12
	F (%)	66.6	64	1.04
Glesby et al.^a^ (DDI) [11]	C_max_ ratio	1.74	1.82	0.96
	AUC_24_ ratio	1.89	1.89	1.0
Menon et al.^b^ (DDI) [12]	C_max_ ratio	1.42 (1.39-1.45)	1.26 (1.11-1.44)	1.13
	AUC_∞_ ratio	2.28 (2.19-2.38)	2.57 (2.31-2.86)	0.89

*IV* intravenous, *DDI* drug–drug interaction

^a^Indinavir/Ritonavir + Amlodipine

^b^Ombitasvir/Paritaprevir/Ritonavir + Dasabuvir + Amlodipine

### Amlodipine-RTV DDI

**Fig. 3:**
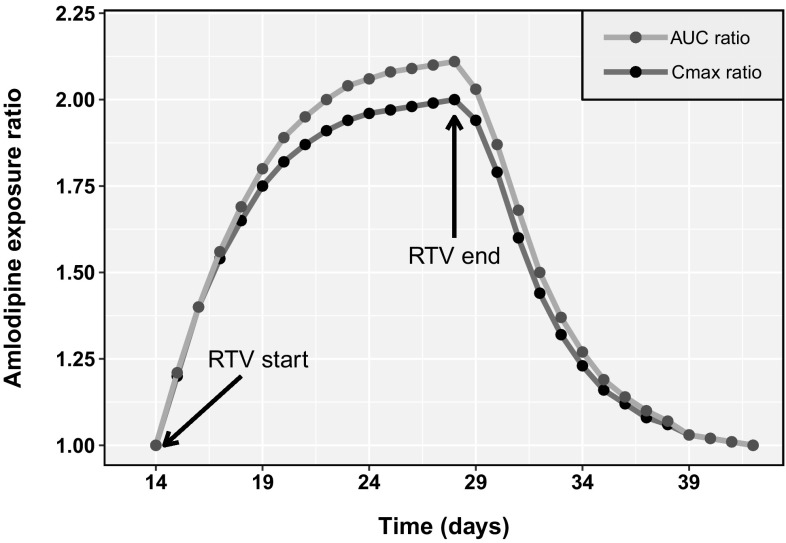
Model prediction of time-based changes in the drug–drug interaction (DDI) magnitude of amlodipine and ritonavir (RTV) over multiple days after amlodipine [2.5 mg once daily (QD)] + RTV (100 mg QD) co-dosing for 14 days (Day 15–Day 28), followed by continuation of amlodipine (2.5 mg QD) alone. This was preceded by amlodipine (2.5 mg QD) dosing for 14 days. The area under the plasma-concentration time curve from time 0 to 24 h (AUC_24_) ratio (orange line) and maximum plasma concentration (C_max_) ratio (blue line) of amlodipine were estimated with respect to steady-state (Day 14) values as reference. (RTV start indicates starting of co-administration of RTV; RTV stop indicates RTV stoppage) (Color figure online)

Simulations of DDI with RTV were compared to clinical data (C_max_ and AUC ratios) from the published reports by Glesby et al. [11] and Menon et al. [12] Table 4 summarizes the PBPK model predictions versus the observed C_max_ and AUC ratios of amlodipine with and without RTV. The PBPK model results met the pre-specified acceptance criteria, with a maximum prediction error of 13% compared with clinical data. The final PBPK model was used to simulate amlodipine plasma PK after co-administration with RTV over time. Figure 3 shows the simulated C_max_ and AUC_24_ ratios over time from day 15 onwards, when RTV dosing was started (simulated dosing schedule discussed in the Methods section). Both the C_max_ and AUC_24_ ratios increased due to CYP3A4 inhibition by RTV and the interaction reached steady state on approximately Day 25. After the last dose of RTV on Day 28, the C_max_ and AUC ratios of amlodipine started to decrease until amlodipine plasma exposures reached baseline levels (C_max_ and AUC ratios of 1.0). As shown in Fig. 3, the DDI ratios reached 1.2 on Day 34, suggesting that 80% of the CYP3A4 inhibition by RTV was resolved within 5 days after the last dose of RTV.

### Amlodipine PD model verification

**Fig. 4:**
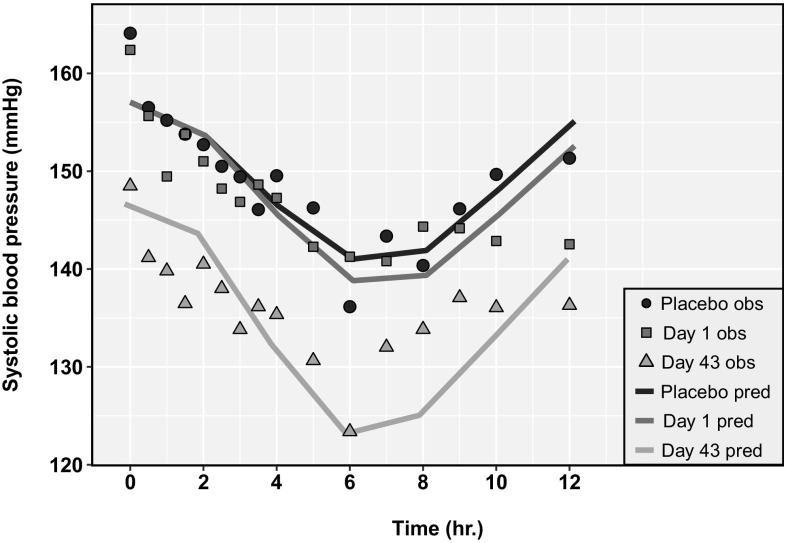
Comparison of model predicted systolic blood pressure (SBP) compared with mean clinical observations from day 1 and day 43 for patients on a 5 mg once daily (QD) regimen of amlodipine or placebo for 6 weeks (data from Donnelly et al. [46]). Symbols represent observed values for placebo (triangle), Day 1 (square), and Day 43 (circle); lines represent predicted values for placebo (black), Day 1 (blue), and Day 43 (orange) (Color figure online)

Figure 4 shows the model-predicted mean SBP compared with the mean observations from 12 hypertensive patients [46]. The predictions are for a ‘population representative’ male subject from the age group of 25–64 years in Simcyp^®^ V15R1. The model predictions successfully captured the circadian variation in SBP and also the decrease in SBP due to amlodipine administration based on visual inspection of the simulated versus observed data. As shown in Fig. 4, the model simulated curves are not in complete agreement with the observations. This might be due to the fact that the simple PD model developed here might not be sufficient to capture effects on blood pressure due to other physiological mechanisms such as circadian effects on blood pressure.

### Amlodipine-RTV PBPK/PD model simulations

**Fig. 5:**
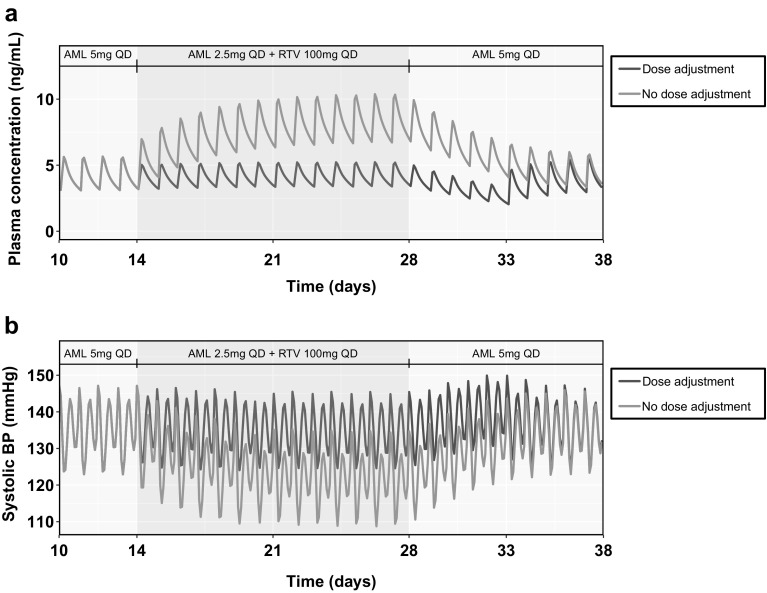
Results for Scenario 1—Predicted plasma concentration of amlodipine (AML) (**a**) and systolic blood pressure (**b**) using a physiologically-based pharmacokinetic (PBPK)/pharmacodynamic (PD) model for a ritonavir (RTV)-amlodipine once daily (QD) dosage regimen with (green line) and without (pink line) amlodipine dose adjustment (Color figure online)

**Fig. 6:**
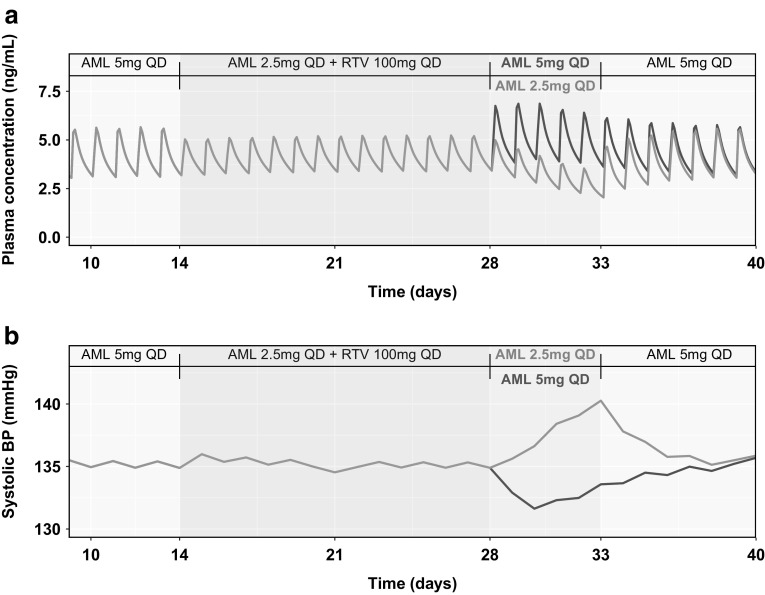
Results for Scenario 2—Predicted plasma concentration of amlodipine (**a**) and daily averaged systolic blood pressure (**b**) using a physiologically-based pharmacokinetic (PBPK)/pharmacodynamic (PD) model for a ritonavir (RTV)-amlodipine dose regimen with a regular amlodipine dose (5 mg once daily [QD]) starting 5 days after RTV stoppage (green line) and a regular amlodipine dose starting immediately after RTV stoppage (pink line) (Color figure online)

The PBPK/PD model was used to simulate the changes in SBP due to the dynamic changes in amlodipine plasma exposures in three separate model simulation scenarios (discussed in detail in the Methods section). Figure 5 shows the results from scenario 1, where the effect of dose adjustment of amlodipine was compared with no dose adjustment. As shown in Fig. 5a, dose adjustment of amlodipine during RTV coadministration was sufficient to maintain amlodipine plasma concentrations at the same level as without RTV. Figure 5b shows that with dose adjustment, SBP was maintained at similar levels as before RTV coadministration. Interestingly, without amlodipine dose adjustment, the predicted SBP dropped to below 110 mmHg at some time points throughout the course of amlodipine administration. Figure 6 shows the results from scenario 2. The simulations suggested that continuing the reduced amlodipine dose (2.5 mg QD) for an 5 additional days results in a lowering of amlodipine plasma concentrations and a corresponding increase in SBP over the 5 days to 149.9 mmHg.

**Fig. 7:**
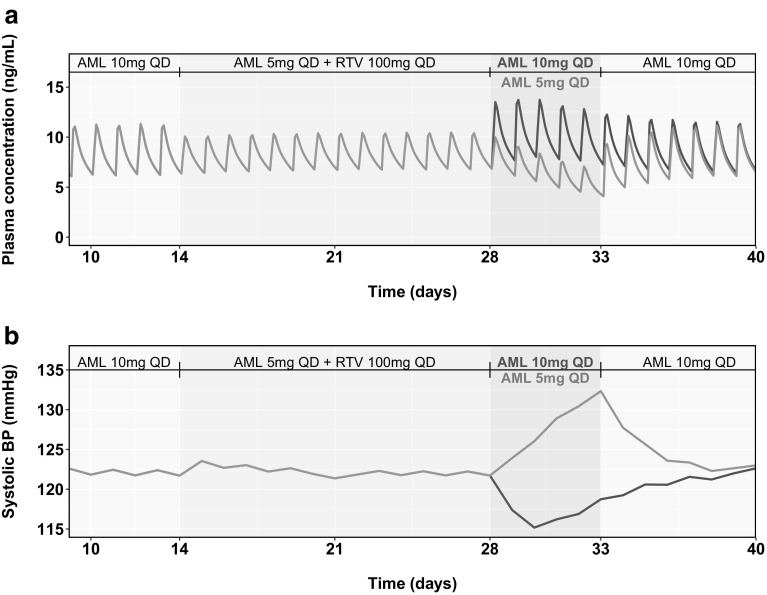
Results for Scenario 3—Predicted plasma concentration of amlodipine (**a**) and daily averaged systolic blood pressure (**b**) using a physiologically-based pharmacokinetic (PBPK)/pharmacodynamic (PD) model for a ritonavir (RTV)-amlodipine dosage regimen with a regular amlodipine dose (10 mg once daily [QD]) starting 5 days after RTV stoppage (green line) and a regular amlodipine dose starting immediately after RTV stoppage (pink line) (Color figure online)

Simulations using the PBPK/PD model suggested that continuing an amlodipine reduced dose of 2.5 mg QD for 5 days after the last dose of RTV results in a slight increase in the daily average SBP to a maximum of 5.8 mmHg above that predicted on the last day of RTV coadministration (Fig. 6b). In contrast, resuming an amlodipine full dose of 5 mg QD immediately after the last dose of RTV results in a decrease of daily average SBP by a maximum of 3.3 mmHg below that predicted on the last day of RTV coadministration (Fig. 6b). Figure 7 shows results from scenario 3, where a similar comparison was done for a higher amlodipine dose level of 10 mg QD. The simulation results suggested that continuing an amlodipine reduced dose (5 mg QD in this case) results in an increase of 10.6 mmHg, while switching immediately to the regular dose results in a decrease of 6.5 mmHg.

## Discussion

A PBPK/PD modeling strategy was used to investigate dose adjustment recommendations for amlodipine during and after co-administration of RTV containing 2- or 3-DAA regimens. Unlike previously published PBPK models for amlodipine [20, 21], which did not confirm the extent and contribution of CYP3A4-mediated clearance, the model developed here is compared against dedicated clinical DDI studies with ritonavir. A PBPK model to be used to predict DDI pertaining to a particular metabolic pathway needs to be adequately qualified and verified for that intended purpose and verification of the fractional contribution of the particular pathway is a critical aspect [14]. This has been pointed out in PBPK guidance documents from regulatory agencies (United Stated Food & Drug Administration [56], European Medicines Agency [57]). The developed PBPK model described the interaction over time between amlodipine and RTV, and simulations suggested that the 5-day window for returning to a full dose of amlodipine was largely due to time-dependent inhibition of CYP3A4 by RTV. Model simulations suggest that continuing with a reduced amlodipine dose for 5 days after a RTV regimen results in a 29% decrease in the average plasma concentration of amlodipine (on the 5th day after the last dose of RTV). On the contrary, resuming a full dose of amlodipine immediately after a RTV regimen results in a 26% increase in the average plasma concentration of amlodipine (on the 3rd day after the last dose of RTV). A local sensitivity analysis of the PBPK model was conducted, and the results (Fig. S6) suggest that plasma C_max_ was most sensitive to the absorption rate constant (*k*_*a*_) and CYP3A4 intrinsic clearance (*CL*_*int*_). This is expected because rate of absorption and CYP3A4 clearance in the GI tract influences the initial plasma concentrations of amlodipine. Volume of distribution (*V*_*d*_) and volume of the single adjusting compartment (*V*_*SAC*_) were also sensitive parameters for C_max_. Sensitivity analysis also suggests that AUC was most sensitive to the clearance processes—CYP3A4 mediated and non-specific clearance. The PBPK model was developed using a 1st order absorption model and a minimal distribution model because amlodipine is considered a BCS class I compound with high solubility and permeability, and plasma and hepatic exposures are sufficient to characterize its efficacy and DDI. One of the limitations of the current 1st order absorption and minimal distribution parts of our PBPK model is that it cannot be used to predict changes in plasma concentrations of amlodipine due to changes in formulation characteristics such as dissolution or to predict distribution to other tissues other than the liver, blood and the GI tract. However, the current amlodipine model serves the intended purpose of predicting DDIs with concomitant CYP3A modulators to inform dose adjustment decisions, and may be expanded to other applications if more data become available to inform for example changes in pharmaceutics characteristics. Model predicted C_max_ for the IV dose was significantly under-predicted compared to the observed data reported by Faulkner et al. [32]. This might be due to the fact that the amlodipine PBPK model developed in Simcyp assumes a minimal distribution model where the drug entering venous blood instantly reaches equilibrium with the entire venous blood volume, while in reality plasma concentrations entering the vein might not equilibrate quickly due to the drug entering low perfusion tissues such as fat/skin/muscle. A full PBPK model could be used to evaluate this under prediction of C_max_, however, it was not selected in this work since amlodipine is not known to have transporter-mediated processes that may influence drug distribution and tissue distribution data and partition coefficients for amlodipine are not available. There were also limitations to the accurate measurement of amlodipine venous blood concentrations at the very early time points after IV dose administration, as pointed out by Faulkner et al. [32], where these early plasma concentration time points were estimated by linear extrapolation and not measured directly, thus adding to the uncertainty in predicting the early plasma concentrations.

The developed PBPK model for amlodipine was further linked to a PD model for SBP. Simcyp^®^ allows the development of mechanistic PBPK models; however, PD modules linked to such PBPK models have traditionally been simple linear or non-linear models. In this article, development of a complex PD model including a diurnal baseline effect has been described utilizing the *Lua* scripting facility in Simcyp^®^. The diurnal model allows one to capture the dynamic oscillation in SBP, which is important for managing cardiovascular risk. For example, the nocturnal decline in SBP and the morning surge are important predictors of various cardiovascular events [52]. Based on the final PBPK/PD model simulations, amlodipine at a reduced dose of 2.5 mg QD may be continued for 5 days after the last dose of RTV, followed by a return to the full dose of 5 mg QD. Alternatively, the full dose of amlodipine may resume immediately, the day after the last dose of RTV. The PBPK/PD model predicted a maximum difference of 3–6 mmHg between the 2 dosing scenarios. However, the difference was greater (10.6 mmHg) for a higher dose level of amlodipine (10 mg QD). Thus model predictions suggest that patients on a higher dose regimen of amlodipine are more vulnerable to changes due to dose adjustment. The difference in average daily SBP also does not reflect diurnal variations in SBP, which may lead to more significant variations. Based on the model predicted dynamic SBP (scenario 2), continuing with a reduced amlodipine dose for 5 additional days leads to a maximum SBP of 149.9 mmHg. Conversely, switching immediately to the regular amlodipine dose leads to a minimum SBP of 119.9 mmHg. For an individual on a regular amlodipine dose of 10 mg QD (scenario 3), the predicted dynamic SBP reaches a low of 98.5 mmHg, which might be a critically low blood pressure. Blood pressure is also dependent on several other factors such as diet, sleep, stress levels and others. The model considers SBP due to amlodipine PK and hence the predictions do not preclude careful clinical monitoring for sudden changes in blood pressure, which might be influenced by other factors and hence difficult to predict using a non-mechanistic PD model. Physiological blood pressure control is a complex process involving the baroreflex loop, autoregulation, or by the renin-angiostenin-aldosterone system [20]. It is affected by food, sleep, stress, and multiple other environmental and genetic factors. The PD model does not consider any of these mechanisms or factors and is therefore limited in its predictive power of actual blood pressure under a variety of conditions.

Effects of disease, age, and co-medications on drug disposition are not additive and cannot always be predicted in a dynamic fashion. Given that amlodipine is commonly co-prescribed with other drugs, the PBPK/PD model developed here may serve as a future tool to simulate various dosing scenarios with and without DDIs with other CYP3A modulators. Another potential utility of the developed PBPK/PD model is that it may also be used to simulate plasma exposures in special populations such as elderly and cirrhotic populations with associated changes in hepatic clearance. The amlodipine PBPK/PD model may be useful in guiding dose adjustment in a variety of scenarios involving amlodipine.

## Electronic supplementary material

Below is the link to the electronic supplementary material.

Supplementary material 1 (PDF 724 kb)
