# Supplementary material for: Guiding dose adjustment of amlodipine after co-administration with ritonavir containing regimens using a physiologically-based pharmacokinetic/pharmacodynamic model
Source: J Pharmacokinet Pharmacodyn. 2018 Feb 9;45(3):443–56. doi: 10.1007/s10928-018-9574-0 (PMC5953987; doi:10.1007/s10928-018-9574-0)
Supplement: Supplementary file 1 — Supplementary material 1 (PDF 724 kb) [file 10928_2018_9574_MOESM1_ESM.pdf]

Supplementary Information

Journal of Pharmacokinetics and Pharmacodynamics

# Guiding Dose Adjustment of Amlodipine after Co-administration with Ritonavir Containing Regimens Using a Physiologically-Based Pharmacokinetic/Pharmacodynamic Model

Dwaipayan Mukherjee[1], Jiuhong Zha[1], Rajeev M. Menon[1], Mohamad Shebley[1]

[1]Clinical Pharmacology and Pharmacometrics, AbbVie Inc., North Chicago, IL, United States

**Correspondence:** Mohamad Shebley, PhD, AbbVie Inc., Clinical Pharmacology and Pharmacometrics, 1 North Waukegan Road, Dept. R4PK, Bldg. AP31-3, North Chicago, IL 60064, Telephone:+1 (847) 938-3662, Email: Mohamad.shebley@abbvie.com

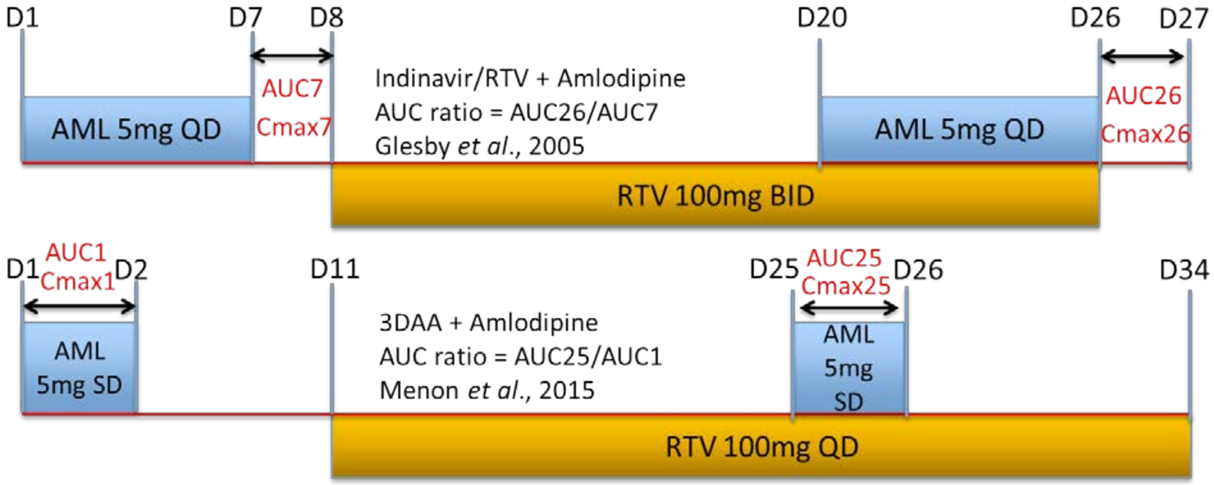


**Figure S1: Clinical study designs of published clinical drug-drug interaction (DDI) studies of amlodipine with ritonavir (RTV)-containing regimens (3DAA refers to the combination regimen of ombitasvir/paritaprevir/RTV + dasabuvir).**

AML, amlodipine; AUC, area under the plasma concentration-time curve; BID, twice daily; $C_{max}$, maximum plasma concentration; D, day; QD, once daily; SD, single dose; AUC1: AUC on day 1 and similarly for others; Cmax1:Cmax on day 1 and similarly for others

```lua
function indirectAlgebraicStep(t, xin, ...)
    -- Effect of amlodipine on systolic blood pressure
    local Po = 148.84 -- Baseline SBP (mmHg)
    local A = 8.245 -- Pressure amplitude (mmHg)
    local om = 0.463 -- cyclic frequency (1/hr)
    local m = -1285.93 -- Pressure slope (mmHg/mu-M)
    local keo = 0.049 -- Decay from effect compartment (1/hr)
    local t1 = 24 * math.floor(t/24) -- Time scaling to get clock time
    xout = Po + A * math.cos(om * (t - t1)) + m * xin * (1 - math.exp(-keo * t))
    return xout
end
```

**Figure S2:** ***Lua*** **script used to define the pharmacodynamic (PD) model for systolic blood pressure within Simcyp V15R1; Parameter definitions are described in the code in green. The equation for** ***xout*** **follows the PD model equation as described in Equation 3 in the main text.**

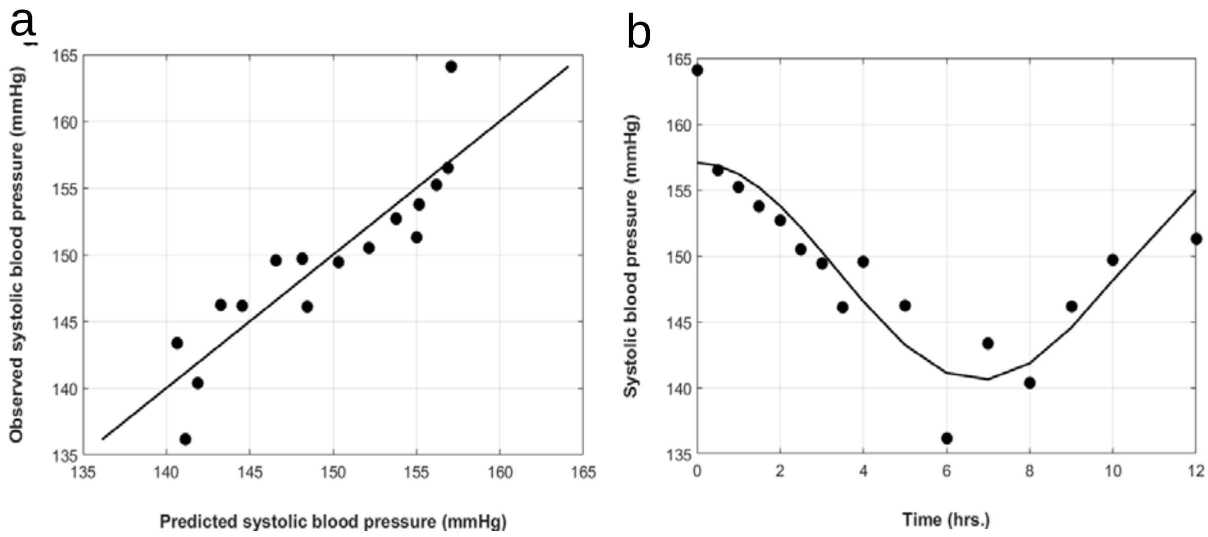


**Figure S3: Diagnostic plots for the placebo model fit: (a) Goodness of fit plot of observed versus predicted systolic blood pressure (black solid line represents the line of unity); (b) visual predictive check plot for the placebo model (black solid line represents model prediction while black dots represent observed data); Observed data from Donnelly** ***et al.*****, 1993**

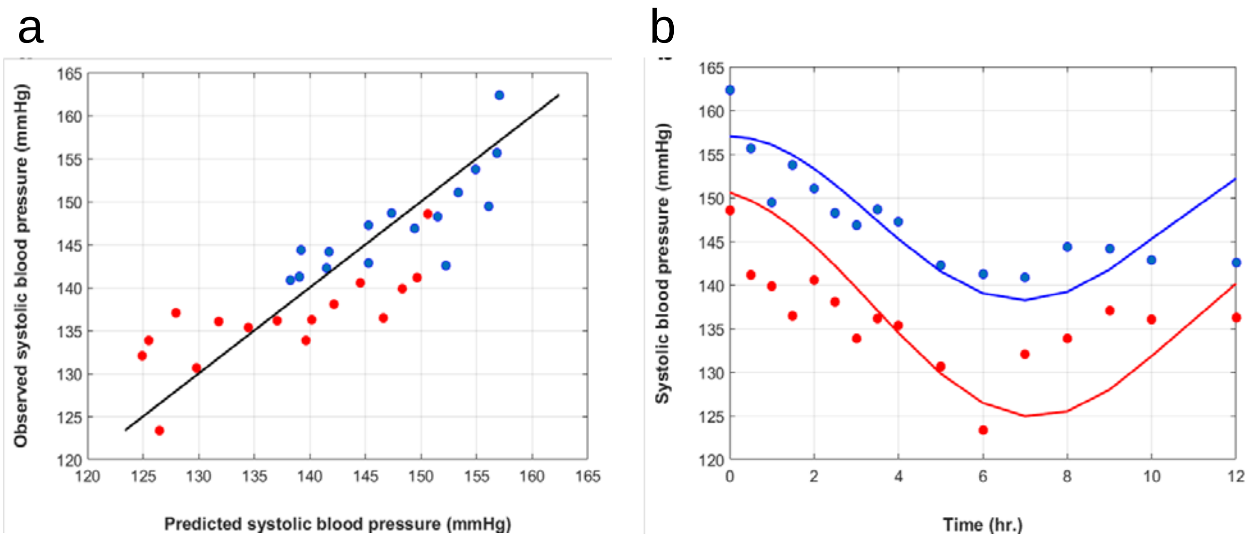


**Figure S4: Diagnostic plots for the complete pharmacodynamic (PD) model fit: (a) Goodness of fit plot of observed versus predicted systolic blood pressure (black solid line represents the line of unity while red and blue dots represent points for day 1 and day 43 respectively); (b) visual predictive check plot for the complete PD model (red and blue colors reresent day 1 and day 43, respectively, while solid lines represent model prediction and dots represent observed data); Observed data from Donnelly *et al*., 1993**

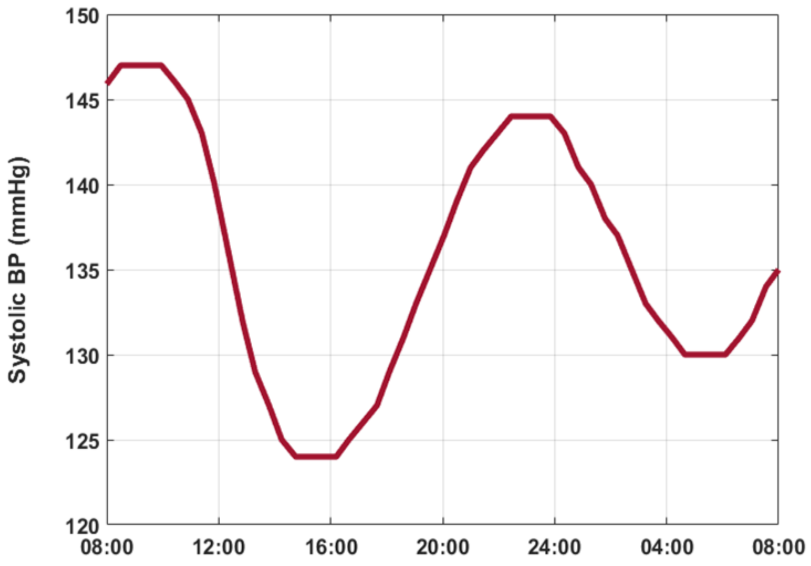


**Figure S5: Model predicted systolic blood pressure over a 24-hour span for a virtual subject on amlodipine 5 mg daily dose at steady-state (after 6 weeks)**

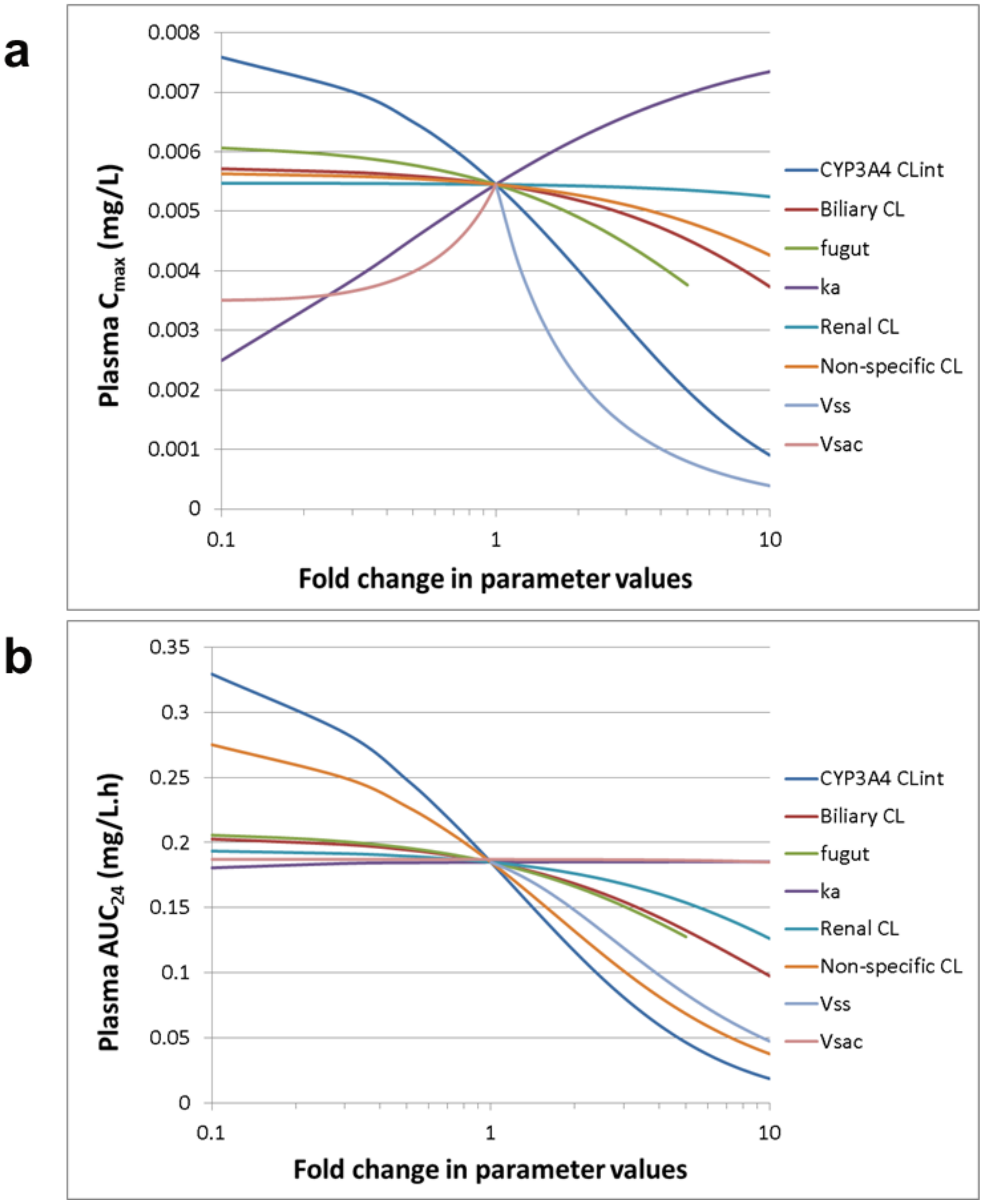


**Figure S6: Results of local sensitivity analysis for 8 parameters in the PBPK model with (a) $C_{max}$ and (b) 24-hour AUC as output variables; Parameter values were changed from their final values in the model by 10 fold in either direction (nominal value represented by 1). y-axis represents (a) maximum plasma concentration ($C_{max}$) and (b) area under the plasma concentration curve integrated over 24 hours.**

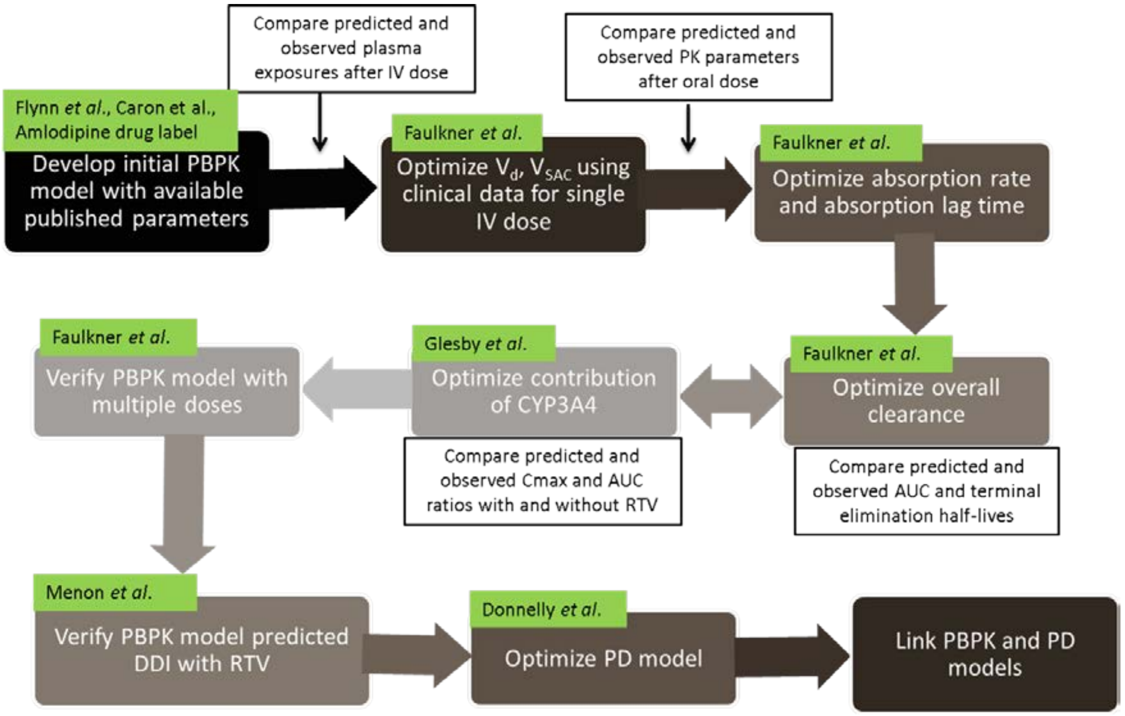


**Figure S7: Flow diagram describing the model development and decision processes along with the data sources used in each step.**

| Assumption | Justification | Implication |
|---|---|---|
| 1st order absorption model | • Model parsimony<br>• BCS class I compound with high rate of absorption<br>• Main motive of model is to predict DDI and not changes in pharmaceutics characteristics | Changes in the oral absorption model does not affect the overall DDI results with RTV |
| Fraction absorbed = 1 | Human mass balance study found same amlodipine recovery in feces after oral and IV administration (Stopher *et al.*, 1988) | Change in fraction absorbed would affect the initial part of the plasma concentrations |
| Minimal PBPK model for distribution | Amlodipine distribution into other tissues is not an important consideration and plasma exposure is sufficient for prediction of efficacy; no known tissue uptake transporters involved | Amlodipine concentration in tissues other than the liver, gut, and blood cannot be predicted by the model |
| Biliary clearance | Amlodipine shown to undergo entero-hepatic recirculation (Rausl *et al*., 2006; Stopher *et al.*, 1988) | No significant effect |
| Minor renal elimination | Renal elimination only 6% (Beresford *et al*., 1988) Renally impaired patients had no change in plasma exposure compared with healthy subjects (Laher *et al*., 1988) | No significant effect |
| Additional non-specific systemic clearance | No other enzymes or transporters have been indicated to be contributing to amlodipine clearance | This ensures agreement of the overall predicted clearance with observed clearance while maintaining known pathway contributions such as CYP3A4 compared with the observed data |
| Systolic blood pressure is the appropriate pharmacodynamic variable | SBP has been indicated to be an important predictor of cardiovascular disease. (Canale *et al*., 1991). It is also more important to control SBP than diastolic blood pressure. (Byyny *et al*., 1997) | This ensures that clinical dose adjustments are related to the key clinical endpoint and outcome |

**Table S1: List of assumptions made during PBPK/PD model development**

| Study | Population | Age (year) | n | Amlodipine Dose |
|---|---|---|---|---|
| Faulkner et al., 1986 | Healthy | 25.8 ± 3.8 | 12 | 10 mg |
| Williams & Cubeddu, 1988 | Healthy | 23 - 34 | 12 | 10 mg |
| Sasaki et al., 2001 | Hypertensive | 78 ± 9 | 8 | 5 mg |
| Rausl et al., 2006 | Healthy | - | 24 | 10 mg |
| Lv et al., 2014 | Healthy (Chinese) | - | 12 | 10 mg |
| Elliott et al., 1988 | Healthy | 72 ± 6.3 | 16 | 5 mg |
| Faulkner et al., 1989 | Healthy | 24.3 ± 3.4 | 12 | 20 mg |
| Stopher et al., 1988 | Healthy | - | 2 | 15 mg |
| Donnelly et al., 1993 | Hypertensive | 25 – 64 | 12 | 5 mg |

**Table S2: Summary of subject demographics for clinical studies with amlodipine**

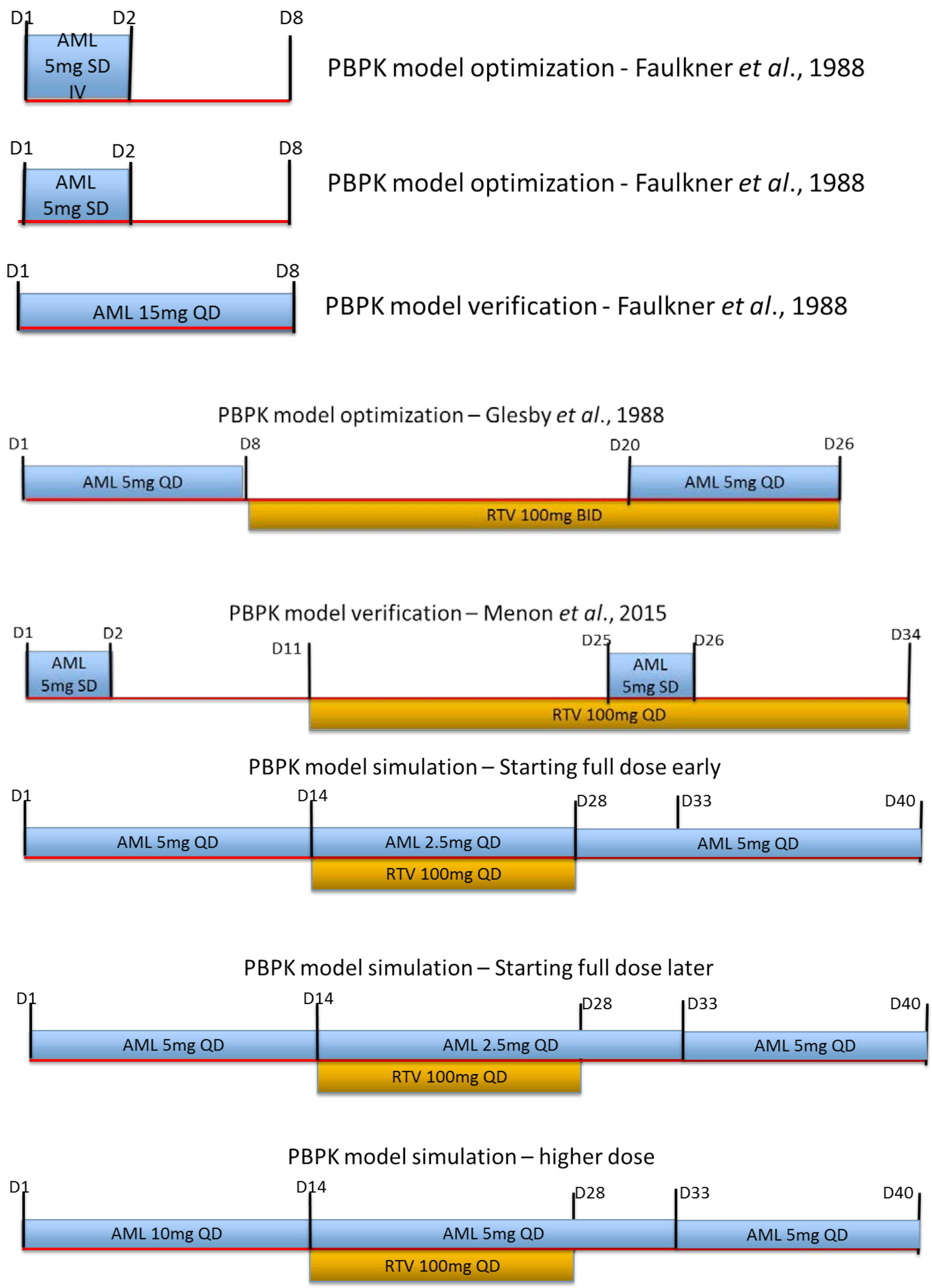


**Figure S8: Simulation trial designs conducted using the final PBPK model in Simcyp**
